# Autoimmune Thyroid Diseases and Thyroid Cancer in Pemphigus: A Big Data Analysis

**DOI:** 10.3389/fmed.2018.00159

**Published:** 2018-05-30

**Authors:** Khalaf Kridin, Mogher Khamaisi, Doron Comaneshter, Erez Batat, Arnon D. Cohen

**Affiliations:** ^1^Department of Dermatology, Rambam Health Care Campus, Haifa, Israel; ^2^Internal Medicine D, Institute of Endocrinology, Diabetes and Metabolism, Rambam Health Care Campus, Haifa, Israel; ^3^Rappaport Faculty of Medicine, Technion-Israel Institute of Technology, Haifa, Israel; ^4^Department of Quality Measurements and Research, Clalit Health Services, Tel Aviv, Israel; ^5^Siaal Research Center for Family Medicine and Primary Care, Faculty of Health Sciences, Ben-Gurion University of the Negev, Beer-Sheva, Israel

**Keywords:** pemphigus, thyroid gland, autoimmunity, comorbid conditions, comorbidity, association, cancer

## Abstract

There is a little consensus regarding the association of pemphigus with autoimmune thyroid diseases. While this association had been confirmed by some observational studies, others had refuted it. We aimed to study the association between pemphigus and Hashimoto's thyroiditis, Grave's disease, and thyroid cancer using a large-scale real-life computerized database. A cross-sectional study was performed to compare pemphigus patients with age-, sex-, and ethnicity-matched control subjects regarding the prevalence of overt thyroid diseases. Chi-square and *t*-tests were used for univariate analysis, and a logistic regression model was used for multivariate analysis. The study was performed using the computerized database of Clalit Healthcare Services ensuring 4.5 million individuals. A total of 1,985 pemphigus patients and 9,874 controls were included in the study. The prevalence of Hashimoto's thyroiditis (12.9 vs. 11.9%; *P* = 0.228), Graves's disease (0.7 vs. 0.7%; *P* = 0.986), and thyroid cancer (0.7 vs. 0.5%; *P* = 0.305) were comparable among patients with pemphigus and control subjects. In sex-stratified analysis, pemphigus associated significantly with Hashimoto's thyroiditis among male patients (OR, 1.36; 95% CI, 1.04–1.79). In multivariate analysis adjusting for potential confounding factors, no independent associations between the conditions were revealed. Study findings were robust to sensitivity analysis that included only patients under pemphigus-specific treatments. In conclusion, Hashimoto's thyroiditis was found to be associated with pemphigus only among male patients, but not among all patients. Physicians treating patients with pemphigus might be aware of this possible association. This study does not provide a clue for an association of pemphigus with Grave‘s disease or thyroid cancer.

## Introduction

Pemphigus is a rare group of organ-specific autoimmune diseases affecting the skin and the mucous membranes. This intraepidermal bullous diseases manifest with vesicles and erosions on the epithelium of mucous membranes and skin, and is often accompanied with increased morbidity and mortality (1, 2). The pathogenesis of pemphigus is characterized by the production of autoantibodies against different proteins of the desmosomes. The binding of these autoantibodies to desmosomal components disrupts intra-epidermal adhesion, leading to acantholysis and intraepithelial blister formation (3, 4). Patients with pemphigus were observed to experience an increased prevalence of several autoimmune diseases (5).

Hashimoto's thyroiditis and Grave's disease are the most widespread autoimmune thyroid disease (AITD). These diseases represent a prototypical organ-specific autoimmune disorder, in which extrinsic factors trigger the development of an immune response directed against thyroid antigens in genetically susceptible individuals (6). AITDs were found to coexist with other comorbid autoimmune disorders, and the concept of an autoimmune diathesis is widely accepted in these diseases (7).

The association between pemphigus and autoimmune thyroid diseases (AITD) is controversial. While several controlled studies revealed a higher prevalence of overt autoimmune thyroid diseases (8–10) and greater positivity of anti-thyroid peroxidase (TPO) among pemphigus patients (11, 12), other studies did not demonstrate a significant association between pemphigus and clinical AITD (11–15).

The objective of the current study is to investigate the association of pemphigus with Hashimoto's thyroiditis, Graves's disease, and thyroid cancer using a large-scale real-life cross-sectional study.

## Methods

### Study design and dataset

This study was designed as a cross-sectional retrospective study utilizing the database of Clalit Healthcare Services (CHS)- the largest managed care organization in Israel, serving a population of approximately 4,500,000 enrollers in 2016. CHS has a database with incessant real-time input from medical, administrative, and pharmaceutical computerized operating systems. The validity of diagnoses in this registry, which are grounded on hospital and primary care physicians and specialists reports, has been found to be of high reliability (16, 17). This database undergoes a regular validation procedures by logistic checks (such as comparing the diagnoses from different sources), as well as by direct validation of the diagnoses by the managing physicians.

The current study was approved by the institutional ethical board of Ben-Gurion University and CHS.

### Study population

Patients were defined as having pemphigus and each one of the thyroid diseases when there was a documented diagnosis of these entities at least twice in the medical records registered by a physician in the community, or when they have been registered in the diagnoses of discharge letters from hospitals. To raise their validity, the definition of Hashimoto's thyroiditis and Grave‘s diseases were based on the relevant ICD-9 codes and prescription of thyroid hormone replacements and anti-thyroid preparations, respectively, for more than 6 months.

A control group of up to 5 controls per each case were selected, matched randomly by age, sex, and ethnicity. The age matching was grounded on the exact year of birth (1-year strata).

### Covariates and outcome measures

Outcome measures were controlled for comorbid conditions as determined using Charlson comorbidity index (18). Outcome measures were also controlled for overutilization of health services, in order to ensure that observed associations were not merely due to ascertainment bias. Healthcare utilization was determined by the number of total visits per individual in the year preceding the diagnosis of pemphigus in cases and the enrollment of control subjects.

Sensitivity analysis was undertaken by repeating all calculations following the inclusion of only cases prescribed “pemphigus-related medications”: systemic corticosteroids or adjuvant immunosuppressive agents (excluding methotrexate) for more than 6 months; or cases prescribed one or more cycles of rituximab.

### Statistical analysis

The distribution of sociodemographic and clinical factors was compared between cases and control subjects using Chi-square test for sex, socioeconomic status, and *t*-test for age. Logistic regression was then used to calculate ORs, and 95% CIs, to compare cases and controls with respect to the specified malignancies. Homogeneity of ORs across strata was tested using Breslow–Day and Tarone's tests. The exact age matching permitted the use of unconditional logistic regression (19). All statistical analysis was performed using SPSS software, version 23 (SPSS, Chicago, IL, USA).

## Results

Our study population comprised 1,985 patients with pemphigus and 9,874 age-, sex-, and ethnicity-matched control subjects (Table 1). The mean (±SD) age at the onset of pemphigus was 72.1 ± 18.5, which is identical to the age of control subjects at their enrollment date. In all, 797 (40.2%) of pemphigus patients were male and similar proportion was observed in controls. No significant differences in ethnic background and socioeconomic status were noted between the two groups. Comorbidity rates, measured by the Charlson index, were higher in cases, with 1,059 (53.4%) patients having severe comorbidity compared with 4,055 (41.1%) in controls (Table 1).

**Table 1:** Descriptive characteristics of the study population.

**Characteristic**	**Patients with pemphigus (*N* = 1985)**	**Controls (*N* = 9874)**	***P*-value**
**AGE, YEARS**			
Mean ± SD	72.1 ± 18.5	72.1 ± 18.5	1.000
Median (range)	77.4 (0–103.0)	77.4 (0–103.1)	
Male sex, *N* (%)	797 (40.2%)	3,962 (40.1%)	0.934
**ETHNICITY**, ***N*** **(%)**			
Jews	1,805 (90.9%)	8,866 (89.8%)	0.136
Arabs	180 (9.1%)	1,008 (10.2%)	
BMI, kg/m^2^ (Mean ± SD)	27.7 ± 6.6	27.9 ± 6.6	0.355
Smoking, *N* (%)	510 (25.7%)	2,758 (27.9%)	0.045
**SES**, ***N*** **(%)**			
Low	634 (31.9%)	3,249 (32.9%)	0.386
Intermediate	830 (41.8%)	4,263 (43.2%)	0.250
High	423 (21.3%)	2,217 (22.5%)	0.241
**CHARLSON COMORBIDITY SCORE**, ***n*** **(%)**			
None (0)	344 (17.3%)	2,636 (26.7%)	< 0.001
Moderate (1–2)	582 (29.3%)	3,183 (32.2%)	0.011
Severe (≥3)	1,059 (53.4%)	4,055 (41.1%)	< 0.001
**HEALTHCARE UTILIZATION**, ***n*** **(%)**			
0 visits	286 (14.4%)	770 (7.8%)	< 0.001
1–12 visits	411 (20.7%)	2,094 (21.2%)	0.248
≥13 visits	1,288 (64.9%)	7,010 (71.0%)	< 0.001

*N, Number; SD, standard deviation; BMI, body mass index; SES, socioeconomic status*.

Table 2 demonstrates the proportions of cases and controls with thyroid diseases stratified by sex and age category. The prevalence of Hashimoto's thyroiditis (12.9 vs. 11.9%) and thyroid cancer (0.7 vs. 0.5%) was slightly higher in patients with pemphigus than in controls, although without reaching the level of statistical significance (*P* = 0.228 and *P* = 0.305, respectively). The prevalence of Grave's disease was comparable among cases and controls (0.7 vs. 0.7%; *P* = 0.986).

**Table 2:** Demographics of cases and controls with overt thyroid diseases.

	**Hashimoto's thyroiditis (*****n*** = **1,434)**, ***n*** **(%)**		**Grave's disease (*****n*** = **78)**, ***n*** **(%)**		**Thyroid cancer (*****n*** = **60)**, ***n*** **(%)**	
**Characteristic**	**Pemphigus (*n* = 1,985)**	**Control (*n* = 9,874)**	**Pemphigus (*n* = 1,985)**	**Control (*n* = 9,874)**	**Pemphigus (*n* = 1,985)**	**Control (*n* = 9,874)**
All (*n* = 11,859)	256 (12.9)	1,178 (11.9)	13 (0.7)	65 (0.7)	13 (0.7)	47 (0.5)
Male (*n* = 4,759)	73 (9.2)	273 (6.9)	2 (0.3)	12 (0.3)	3 (0.4)	8 (0.2)
Female (*n* = 7,100)	183 (15.4)	905 (15.3)	11 (0.9)	53 (0.9)	10 (0.8)	39 (0.7)
**AGE CATEGORY (YEARS)**						
< 40 (*n* = 872)	5 (3.5)	12 (1.6)	0 (0.0)	1 (0.1)	0 (0.0)	3 (0.4)
40–59 (*n* = 1,768)	26 (8.8)	87 (5.9)	2 (0.7)	5 (0.3)	0 (0.0)	8 (0.5)
60–79 (*n* = 4,121)	102 (14.8)	404 (11.8)	5 (0.7)	17 (0.5)	7 (1.0)	24 (0.7)
≥80 (*n* = 5,098)	123 (14.3)	675 (15.9)	6 (0.7)	42 (1.0)	6 (0.7)	12 (0.3)

*n, Number. The figures inside the brackets represent the percentage of the positive cases of all individuals in the same strata*.

Table 3 presents the results of univariate and logistic regression models and summarizes ORs for AITD and thyroid cancer among patients with pemphigus. In univariate analysis, no statistically significant association was established between pemphigus and Hashimoto's thyroiditis (OR, 1.09; 95%CI, 0.95–1.22), Grave's disease (OR, 0.99; 95% CI, 0.55–1.81), or thyroid cancer (OR, 1.38; 95% CI, 0.74–2.55). However, in sex-stratified analysis, Hashimoto's thyroiditis was significantly associated with pemphigus among men (OR, 1.36; 95% CI, 1.04–1.79).

**Table 3:** The association between pemphigus and thyroid diseases.

**Disease**	**Pemphigus (*n* = 1,985), *n* (%)**	**Controls (*n* = 9,874), *n* (%)**	**OR (95% CI)**	**Univariate *P*-value**	**Male-specific OR (95%CI)**	**Female-specific OR (95%CI)**	**Sensitivity analysis OR (95%CI)**	**Adjusted OR (95%CI)^a^**	**Adjusted OR (95%CI)^b^**
Hashimoto's thyroiditis	256 (12.9)	1,178 (11.9)	1.09 (0.95–1.26)	0.228	**1.36 (1.04–1.79)**	1.01 (0.85–1.20)	1.07 (0.91–1.22)	1.00 (0.86–1.16)	1.01 (0.87–1.17)
Grave's disease	13 (0.7)	65 (0.7)	0.99 (0.55–1.81)	0.986	0.83 (0.19–3.71)	1.03 (0.54–1.98)	1.01 (0.57–1.84)	0.93 (0.51–1.70)	0.88 (0.48–1.62)
Thyroid cancer	13 (0.7)	47 (0.5)	1.38 (0.74–2.55)	0.305	1.87 (0.49–7.05)	1.28 (0.64–2.57)	1.34 (0.68–2.66)	1.17 (0.63–2.17)	1.23 (0.66–2.30)

*n, Number; OR, odds ratio; CI, confidence interval*.

a *Adjusted for Charlson score*.

b *Adjusted for healthcare utilization. Bold indicates significant values*.

We further performed a sensitivity analysis, including only pemphigus patients who were prescribed one of the following “pemphigus-related treatments”: systemic corticosteroids or adjuvant immunosuppressive agents (azathioprine, mycophenolate mofetil, cyclophosphamide); or cases prescribed one or more cycles of rituximab. The association of pemphigus with the three aforementioned thyroid diseases lacked statistical significance also in this analysis (Hashimoto's thyroiditis: OR, 1.07; 95% CI, 0.91–1.22; Grave's disease: OR, 1.01; 95% CI, 0.57–1.84; thyroid cancer: OR, 1.34; 95% CI, 0.68–2.66; Table 3).

In a multivariate logistic regression model, no association was observed between pemphigus and Hashimoto‘s thyroiditis, Grave‘s disease, and thyroid cancer after adjusting for comorbidities and over-utilization of healthcare services (Table 3). The multivariate analysis was performed despite the lack of a significant outcome in univariate analysis in order to control for putative negative confounders that may interfere with the association between the entities by suppression effect (20).

## Discussion

This large-scale study is the first population-based study aiming to investigate the associations between pemphigus and autoimmune thyroid diseases and thyroid cancer. Our findings revealed that Hashimoto's thyroiditis was found to be associated with pemphigus among male patients, whereas no association was observed between pemphigus and Grave‘s disease and thyroid cancer.

“Autoimmune diathesis” is a concept alleging that individuals affected by an autoimmune disease are more susceptible to develop other autoimmune disease (21–23). In accordance with the findings of controlled observational studies, pemphigus was found to associate with other autoimmune diseases, including rheumatoid arthritis (9, 10), type I diabetes mellitus (10), Sjögren's syndrome (15), systemic lupus erythematosus (15), alopecia areata (15), ulcerative colitis (24), and myasthenia gravis (2). In addition, both Hashimoto's thyroiditis and Grave's disease are known to cluster with a wide range of autoimmune disorders (6, 25).

With regard to the link between pemphigus and AITD, there is a little consensus, as results from the fact that different studies are inconsistent. Leshem et al. (9) reported that the prevalence of AITD was greater among 110 patients with pemphigus as compared to 969 of their first-degree relatives. The diagnosis of AITD, in this study, was determined differentially using serological analyses in cases and using questionnaires in controls. Parameswaran et al. (10) found that the prevalence of AITD was 6-fold greater among pemphigus patients as compared to the general population. Similarly, the definition of outcome measure was differential; the prevalence of AITD was determined according to a questionnaire in cases and obtained from the published literature and the Centers for Disease Control and Prevention for the general population. In a Turkish study including 80 patients and 80 control subjects, the frequency of anti-TPO antibodies and the prevalence of Hashimoto‘s thyroiditis were significantly higher among cases (8).

Conversely, two small case-control studies including 15 and 22 patients with pemphigus demonstrated higher detection rate of anti-TPO antibodies in the sera of patients with pemphigus relative to controls. However, no differences were noted between cases and controls in terms of clinical thyroid diseases including AITDs (11, 12). Another Iranian case-control study comprising 75 patients and 65 controls found no significant differences between cases and control subjects whether in serum positivity to anti-TPO and anti-thyroglobulin antibodies or in the prevalence of Hashimoto‘s thyroiditis (13).

Apparently, the conclusiveness of the aforementioned studies is severely hampered by the small size of the cohorts utilized. By using one of the largest pemphigus cohorts reported so far, we overcame one of the main drawbacks of previous studies, which hinder a better understanding of pemphigus associations and comorbidities. Lack of association with Hashimoto‘s thyroiditis among female patients and with Grave‘s disease across the entire cohort align with the findings of another population-based Taiwanese study aiming to evaluate the prevalence of comorbid autoimmune disease in pemphigus patients (15). In this large-scale study including 1,998 patients, AITDs were not found to associate with pemphigus (15).

### Strengths and limitations

The large sample size provides sufficient power to exclude chance as the basis for the findings, and enables a precise estimation of the association between uncommon conditions which have otherwise been unavailable. The population-based setting minimizes the probability of selection bias. The study limitations include the lack of data concerning the immunopathological subtype, clinical features, and severity of pemphigus. The utilization of routinely collected data interferes with a direct validation of diagnoses; however, it is improbable that significant misclassification will have meaningfully affected our findings. The diagnoses of both pemphigus and thyroid diseases in our study are very reliable, because pemphigus in Israel is diagnosed in secondary and tertiary care facilities, relying on skin biopsies, direct and indirect immunofluorescence (26), and because the chronic diseases registry of CHS undergoes continuous validation process including verifying diagnoses according to laboratory analyses, making the diagnosis of AITD more precise. To further refute this misclassification, we performed a sensitivity analysis to validate the diagnosis of pemphigus among cases, and we relied on the prescription of relevant treatments to define the diagnosis of AITD.

In conclusion, the present study demonstrates that pemphigus may be associated with Hashimoto‘s thyroiditis only among male patients. Physicians treating patients with pemphigus might be aware of this possible association. This study does not provide a clue for an association of pemphigus with Grave's disease or thyroid cancer.

## Author contributions

KK, AC contributed to study concept and design. KK drafted the manuscript. KK, EB, and DC contributed to the acquisition, analysis, and interpretation of data. AC, MK supervised the study. All authors take responsibility for the integrity of the data and the accuracy of the data analysis.

### Conflict of interest statement

The authors declare that the research was conducted in the absence of any commercial or financial relationships that could be construed as a potential conflict of interest.
